# Mangiferin Improves Hepatic Lipid Metabolism Mainly Through Its Metabolite-Norathyriol by Modulating SIRT-1/AMPK/SREBP-1c Signaling

**DOI:** 10.3389/fphar.2018.00201

**Published:** 2018-03-07

**Authors:** Jian Li, Mengyang Liu, Haiyang Yu, Wei Wang, Lifeng Han, Qian Chen, Jingya Ruan, Shaoshi Wen, Yi Zhang, Tao Wang

**Affiliations:** ^1^Tianjin State Key Laboratory of Modern Chinese Medicine, Tianjin University of Traditional Chinese Medicine, Tianjin, China; ^2^Houston Methodist Hospital, Houston, TX, United States; ^3^Key Laboratory of Pharmacology of Traditional Chinese Medical Formulae, Ministry of Education, Tianjin University of Traditional Chinese Medicine, Tianjin, China

**Keywords:** SIRT-1, AMPK, mangiferin, norathyriol, hepatic lipid metabolism, SREBP-1c

## Abstract

**Objective:** Mangiferin (MGF) is a natural xanthone, with regulation effect on lipid metabolism. However, the molecular mechanism remains unclear. We purposed after oral administration, MGF is converted to its active metabolite(s), which contributes to the effects on lipid metabolism.

**Methods:** KK-A^y^ mice were used to validate the effects of MGF on lipid metabolic disorders. Liver biochemical indices and gene expressions were determined. MGF metabolites were isolated from MGF administrated rat urine. Mechanism studies were carried out using HepG2 cells treated by MGF and its metabolite with or without inhibitors or small interfering RNA (siRNA). Western blot and immunoprecipitation methods were used to determine the lipid metabolism related gene expression. AMP/ATP ratios were measured by HPLC. AMP-activated protein kinase (AMPK) activation were identified by homogeneous time resolved fluorescence (HTRF) assays.

**Results:** MGF significantly decreased liver triglyceride and free fatty acid levels, increased sirtuin-1 (SIRT-1) and AMPK phosphorylation in KK-A^y^ mice. HTRF studies indicated that MGF and its metabolites were not direct AMPK activators. Norathyriol, one of MGF’s metabolite, possess stronger regulating effect on hepatic lipid metabolism than MGF. The mechanism was mediated by activation of SIRT-1, liver kinase B1, and increasing the intracellular AMP level and AMP/ATP ratio, followed by AMPK phosphorylation, lead to increased phosphorylation level of sterol regulatory element-binding protein-1c.

**Conclusion:** These results provided new insight into the molecular mechanisms of MGF in protecting against hepatic lipid metabolic disorders via regulating SIRT-1/AMPK pathway. Norathyriol showed potential therapeutic in treatment of non-alcoholic fatty liver disease.

## Introduction

Sirtuins (SIRT), NAD^+^-dependent lysine deacetylases, are important modulators of energy metabolism and stress resistance (Hirschey, 2011). SIRT-1 is known to be involved in fatty acid synthesis, oxidation, and adipocyte generation (Zhang et al., 2017). In the process of energy stress, along with increased level of NAD^+^, SIRT-1 can directly deacetylates LKB1 and increases its cytoplasmic/nuclear ratio (Lan et al., 2008). Activation of LKB1 can then promote its downstream AMPK phosphorylation. In addition, when AMP level increases, it binds to AMPKγ subunit-cystathionine synthase of alanine and threonine, triggering structural changes and phosphorylation of AMPKα Thr172 residue, and eventually activating AMPK (Viollet et al., 2009). AMPK activation will suppress SREBP-1c cleavage and nuclear translocation, inhibit ACC activity and malonyl CoA production, resulting in fat synthesis suppression and fatty acid oxidation stimulation (Schultze et al., 2012). Thus, SIRT-1/LKB1/AMPK axis controls the whole-body energy-consumption and energy-production.

Liver is a central metabolic organ that regulates essential functions of lipid homeostasis. SIRT-1 plays an important role in TG metabolism in the liver, and increasing evidences suggest an inverse correlation between SIRT-1 levels and non-alcoholic fatty liver disease (NAFLD) (Mariani et al., 2015; Guo et al., 2017). Liver specific deletion of SIRT-1 contributes to increased inflammation, endoplasmic reticulum stress and hepatic steatosis (Purushotham et al., 2009). While SIRT-1 over-expression or activation promotes lipolysis, fat loss and remarkably protects high-fat diet induced obese mice from liver steatosis (Pfluger et al., 2008; Yoo et al., 2017). Previous reports indicate that many natural products exert regulatory effects on liver lipid metabolism through activation of SIRT-1, among which are resveratrol (Ahn et al., 2008), alpha-lipoic acid (Chen et al., 2012), troxerutin (Zhang et al., 2014), and salvianolic acid B (Zhang et al., 2017).

Mangiferin is a xanthone glucoside extracted from the leaves of *Mangifera indica* or the root of *Anemarrhena asphodeloides*, which has effects on reducing lipid accumulation by inhibiting hepatic TG biosynthesis in fructose induced fatty liver rat model (Xing et al., 2014). Oral administration of MGF induces AMPK phosphorylation and the expression levels of its downstream proteins, including fatty acid translocase and carnitine palmitoyl transferase 1 (CPT1) in hyperlipidemic rats (Niu et al., 2012). Consistent with these study, our previous results showed MGF and its homologues up-regulated AMPK at mRNA levels, suppressed the gene expressions of SREBP-1c, FAS, ACC, and HSL in 3T3-L1 preadipocytes (Zhang et al., 2013). These results suggest that the regulatory effects of MGF on metabolic disorders were related to AMPK signaling pathway. However, the detail mechanisms are still not well investigated.

One interesting phenomenon of MGF is its low bioavailability. Pharmacokinetic research reveals that the relative bioavailability of oral MGF is 1.2% in rats (Han et al., 2010). However, the effects of MGF on improving lipid metabolism are remarkable with such a low absorption rate. We hypothesized that after metabolized by either intestinal flora or the liver metabolizing enzymes, MGF was converted to its active metabolite(s) with higher membrane permeability and/or target affinity, which contributed to the remarkable effects on lipid metabolism. Targeting at a signaling cascade is another possibility to explain this phenomenon.

We found a significant up-regulation of SIRT-1 expression in liver upon MGF oral treatment, which highly suggested that SIRT-1 signaling pathway may mediate the effects of MGF. In the current study, with combined pharmacological and chemical approaches, we partly clarified the *in vivo* active metabolites of MGF, and elucidated the underlying mechanism of MGF in regulating hepatic lipid metabolism.

## Materials and Methods

### Reagents

MGF was isolated from Mango leaves as our previous report (Zhang et al., 2013), purity of which was more than 95% determined by a HPLC method. 5-Aminoimidazole-4-carboxyamide-ribonucleoside (AICAR) and Compound C (Dorsomorphin) were purchased from Sigma-Aldrich (St. Louis, MO, United States). STO-609 was obtained from Calbiochem (Merck, San Diego, CA, United States). Assay kits of GLU and TG were from BioSino Bio-Technology and Science Inc. (Beijing, China). FFA assay kit was from Wako Pure Chemical Industries, Ltd. (Osaka, Japan). Nuclear and cytoplasmic extraction reagents and Pierce BCA protein assay kit were from Thermo Fisher Scientific (Waltham, MA, United States). Rabbit anti-beta-actin, anti-AMPKα, anti-*p*-AMPKα-Thr172, anti-ACC, anti-*p*-ACC-Ser79, anti-SREBP-1c, anti-ATGL, anti-CaMKK, anti-LKB1, and anti-HSL antibodies were obtained from Abcam plc. (Cambridge, MA, United States), anti-*p*-HSL-Ser563, anti-CPT1, anti-*p*-SREBP-1c-Ser372, anti-*p*-CaMKK-Ser511, and anti-*p*-LKB1-Ser428 antibodies were from Cell Signaling Technology, Inc. (Beverly, MA, United States). Rabbit anti-lamin-B1 antibody was obtained from GeneTex, Inc. (Irvine, CA, United States). Acetyl-lysine antibody was from ImmuneChem Pharmaceuticals Inc. (Burnaby, BC, Canada). Protein A/G Plus-agarose beads and normal rabbit IgG were obtained from Santa Cruz Biotechnology Inc. (Santa Cruz, CA, United States). All other chemicals were purchased from Sigma-Aldrich (St. Louis, MO, United States) except as indicated.

### *In Vivo* Studies

All animal experiments were approved by Tianjin University of Traditional Chinese Medicine Committee on Use and Care of Animals (TCM-LAEC20170027). Male C57BL/6 and KK-A^y^ mice (6 weeks of age) were purchased from Beijing HFK Bioscience Co., Ltd. (Beijing, China). Mice were housed in a specific pathogen-free grade condition and maintained on a 12–12 h light dark cycle with free access to regular chow and water. The mice were acclimatized for 7 days before treatment. The treatment groups of animals tested are shown in **Table 1**. MGF was administrated orally in 1% gum acacia at a volume of 0.1 ml/10 g BW. The treatment of compound C [dissolved in 5% (*v*/*v*) DMSO/saline] was received every 2 days by intraperitoneal injection at a dosage of 10 mg/kg BW. All the remainders were received equal volumes of vehicle in the same way.

**Table 1:** Experimental design tested animals.

Group name	Animal type	Number	Administration	
			(MGF) (mg/kg/d)	CC (mg/kg/2d)
NC	C57BL/6	9	0	0
NC + CC		9	0	10
DC		9	0	0
DC + CC		9	0	10
200		9	200	0
200 + CC		9	200	10
100	KK-A^y^	9	100	0
100 + CC		9	100	10

*Male KK-A^*y*^ mice were randomly divided into six groups: diabetic control (DC), 100 and 200 mg/kg treatment group, and corresponding compound C co-treatment groups. Male C57BL/6 wild type mice were used as normal control (NC) or NC treated with compound C (CC)*.

During the experimental period of 4 weeks, BW of each animal was measured every 3 days and orbital venous blood was collected every 1 week to determine the non-fasting serum GLU, TG, and FFA levels. After the last administration, all the mice were fasted for 12 h with free access to water. Then the fasting bloods were collected and animals were sacrificed with a lethal dose of anesthetic. Liver and fat (including mesentery fat, perinephric fat, epididymal fat, and interscapular brown and white fat) were excised immediately, rinsed in saline solution, blotted on filter paper, weighed, frozen in liquid nitrogen, and stored at -80°C until analysis.

Liver total lipid content was extracted as previously described (Chen et al., 2014). The total lipid extraction was then loaded onto a thin-layer chromatography (TLC) plate using *n*-heptane/isopropyl ether/acetic acid (60:40:3, *v*/*v*/*v*) as develop solvent. Lipid bands were visualized by immersing the plate in a 10% (*v*/*v*) sulfuric acid-ethanol solution and heated at 120°C for 2 min. The band intensity was measured using ImageJ software (National Institutes of Health, Bethesda, MD, United States).

SD rats (220 ± 10 g; Vital River Laboratory Animal Technology Co., Ltd., Beijing, China) were kept in an environmentally controlled breeding room for 7 days before experimentation, fed with standard laboratory food and water. Animals’ treatment, urine, and liver metabolites analysis were carried out as described in Supplementary Data Sheet 1.

### Histological and Immunohistochemical Staining

For analysis of hepatic lipid droplet accumulation, frozen sections of liver tissues were stained with Oil Red O as described previously with slightly modified (Chen et al., 2014). Liver tissue frozen sections were fixed for 30 min with 10% formalin. 5% Oil Red O stain was applied for 30 min, and then washed three times with 70% ethanol and observed under a light microscope.

For H&E staining, tissues were fixed in 10% buffered formalin, embedded in paraffin wax, cut into about 5-μm-thick sections, mounted on glass slides and stained with H&E after dehydration. For immunohistochemistry (IHC), liver sections were stained with anti-*p*-AMPKα-Thr172 (1:200 dilution) antibodies at 4°C overnight, followed by incubation with secondary antibody and visualized by using a VECTASTAIN^®^ ABC kit (Vector Laboratories Inc., Burlingame, CA, United States). For semi-quantitative analysis, the percentage of the staining intensity (0, negative; 1, weak positive; 2, moderate positive; and 3, strong positive) were recorded and assessed by histoscore (H-score) system (Azim et al., 2015), which was calculated using the following formula:

$$\begin{array}{c} H-Score\;=\;\Sigma (\mathrm{Pi}\;\times \;I)\;=\;(\mathrm{percentage}\;\mathrm{of}\;\mathrm{cells}\;\mathrm{of}\;\mathrm{weak}\;\mathrm{intensity}\;\times \;1)\;+\;(\mathrm{percentage}\;\mathrm{of}\;\mathrm{cells}\;\mathrm{of}\;\mathrm{moderate}\;\mathrm{intensity}\;\times \;2)\;+\;(\mathrm{percentage}\;\mathrm{of}\;\mathrm{cells}\;\mathrm{of}\;\mathrm{strong}\;\mathrm{intensity}\;\times \;3) \end{array}$$

### Cell Culture

The HepG2 cell line (SCSP-510) was obtained from the Chinese Academy of Sciences (Shanghai, China). The cells were maintained in MEM medium containing 10% (v/v) FBS, 1% antibiotic/antimycotic, 1% glutamax, 1% non-essential amino acids, and 1% sodium pyruvate solution. Cells were switched to serum-free medium at ∼90% confluence to receive treatment.

### Inhibition of LKB1 Expression in HepG2 Cells by siRNA

Small interfering RNA of LKB1 and negative control were chemically synthesized by GenePharma Co., Ltd. (Shanghai, China). The siRNA sequence for targeting LKB1 was 5′-CCAACGUGAAGAAGGAAAUTT-3′. As negative control, a siRNA sequence targeting luciferase was used: 5′-UUCUCCGAACGUGUCACGUTT-3′. Transfection of control and LKB1 siRNA (100 nmol/l for each well of 48-well plates) was performed using Lipofectamine-2000 siRNA Transfection Reagent (Invitrogen, United States), according to the manufacturer’s instructions.

### Sodium Oleate (SO) Induced TG Accumulation in HepG2 Cells

The induction of intracellular TG accumulation model by SO was performed according to the methods described previously (Gao et al., 2014). Briefly, cells were seeded on 48-well plates for 24 h, followed by treated with or without SO (200 μmol/l) in the presence or absence of indicated concentrations of MGF (5, 25, 50, and 100 μmol/l) or its metabolites (1, 5, 25, and 50 μmol/l). Orlistat (5 μmol/l) was used as a positive control. AMPK inhibitor Compound C (10 μmol/l), AMPK activator AICAR (100 μmol/l), CaMKK inhibitor STO-609 (10 μmol/l) were used for mechanism researches, respectively. After 48 h treatment, the intracellular TG content was determined with a commercial TG kit at 492 nm after cell lysis. The protein amount was simultaneously determined using a protein assay kit at 562 nm.

### Nile Red Staining

Nile Red staining was used for lipid accumulation state analysis in HepG2. The stock solution was made in acetone (1 mg/ml) and stocked at -20°C in dark. Seeded cells in 24-well plates were transferred to Nile Red at a final concentration of 1 μg/ml and incubated at room temperature for 30 min in a dark condition. After staining, an inverted fluorescence microscope (Nikon, Melville, NY, United States) was used for fluorescent imaging capture and a FACScan Flow Cytometer (BD Biosciences, San Jose, CA, United States) was used for quantitative assessment.

### Western Blotting and Immunoprecipitation (IP)

Protein isolation and Western blotting were performed as described previous (Zhang et al., 2013). For IP study, cell lysates were incubated with 1 μg anti-LKB1 antibody at 4°C for overnight under constant shaking. Non-specific rabbit IgG was used as control. Precipitation was performed by adding 40 μl of immobilized protein A/G Plus-agarose beads under constant shaking for 2–4 h at 4°C. Beads were then centrifuged at 3,000 rpm at 4°C for 5 min, washed 3–4 times with RIPA lysis buffer and finally mixed with 40 μl SDS–PAGE sample loading buffer and boiled for 5 min. 20 μl of the immunoprecipitated proteins were separated on a 10% SDS–PAGE followed by immunoblotting against acetyl-lysine antibody.

### Real-Time PCR Analysis

RNA isolation, cDNA synthesis and real-time PCR analysis were performed as described previously (Liu et al., 2015). The primer sequences used for real-time PCR were shown in **Table 2**. Results were presented as levels of expression relative to those of controls after normalization to β-actin using the 2^–∆∆C^_T_ methods.

**Table 2:** Gene-specific primers used for quantitative real-time PCR.

Gene	Sequence
SREBP-1c (human)	Forward: 5′-ACAGTGACTTCCCTGGCCTAT-3′
	Reverse: 5′-GCATGGACGGGTACATCTTCAA-3′
FASN (human)	Forward: 5′-ACAGCGGGGAATGGGTACT-3′
	Reverse: 5′-GACTGGTACAACGAGCGGAT-3′
CPT1α (human)	Forward: 5′-ATCAATCGGACTCTGGAAACGG-3′
	Reverse: 5′-TCAGGGAGTAGCGCATGGT-3′
β-actin (human)	Forward: 5′-CTGGAACGGTGAAGGTGACA-3′
	Reverse: 5′-AAGGGACTTCCTGTAACAATGCA-3′

*SREBP-1c: sterol regulatory element binding protein-1c; CPT1: carnitine palmitoyl transferase 1; FASN: fatty acid synthase.*

### Analysis of AMP and ATP Levels in HepG2 Cells

Phosphorylated adenosine nucleotides were quantified by HPLC as literature (Obis et al., 2014) with some modifications. HepG2 cells were disposed and collected as this method for HPLC analysis. Chromatography was performed at a flow rate of 1 ml/min on an Agilent 1260 LC system equipped with a VWD detector and a Nacalai tesque Cosmosil 5C18-MS-II column (4.6 ID × 150 mm, 5 μM particle size). Buffer A contained 150 mmol/l KH_2_PO_4_ (pH was adjusted to 6.5 by KOH) and buffer B was composed of 50% (*v*/*v*) acetonitrile and methanol. Injection volume was 30 μl, flow rate was 1 ml/min, detection wavelength was 254 nm, and column temperature was 20°C. Gradient flow program was as follows: 0–3 min: 100% A; 3–10 min: 90% A and 10% B; 10–12 min: 100% A.

### Data Analysis

Data were generated from at least three independent experiments, Values were expressed as mean ± SEM. All the grouped data were statistically performed with SPSS 11.0. Significant differences between means were evaluated by one-way analysis of variance (ANOVA) and Tukey’s Studentized range test was used for post hoc evaluations. *p* < 0.05 was considered to indicate statistical significance.

## Results

### MGF Reduced Hyperlipidemia and Hyperglycemia in KK-A^y^ Mice

**FIGURE 1:**
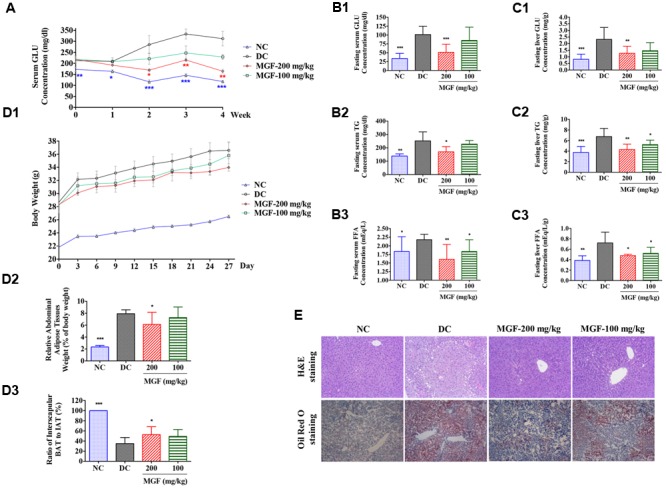
Regulation effect of MGF on hepatic lipid accumulation in KK-A^y^ mice. **(A)** Administration of mangiferin (MGF) suppressed the non-fasting serum glucose (GLU) contents. **(B)** Administration of MGF suppressed the fasting serum GLU **(B1)**, triglycerides (TG) **(B2)**, and Free fatty acid (FFA) **(B3)** contents. **(C)** Administration of MGF suppressed the non-fasting liver GLU **(C1)**, TG **(C2)** and FFA **(C3)** contents. **(D)** Administration of MGF had no obvious effect on body weight (BW) **(D1)**, but effectively suppressed the abdominal adipose tissue weight **(D2)**, and increased the ratio of interscapular brown adipose tissue (BAT) and white adipose tissue (WAT) **(D3)**. **(E)** Liver tissues were stained with hematoxylin and eosin (H&E) or Oil Red O to visualize lipid contents. Pictures were captured under a microscope (40×). Values represent the mean ± SEM of determinations (*n* = 9). ^∗∗∗^*p* < 0.001, ^∗∗^*p* < 0.01, ^∗^*p* < 0.05 versus diabetic control (DC) group.

To investigate the *in vivo* regulation effect of MGF on lipid metabolism, KK-A^y^ mice were treated with MGF (100 or 200 mg/kg/day) for 4 weeks. From 2 weeks to the end of administration, compared with control group, serum non-fasting GLU decreased significantly in 200 mg/kg/d MGF treated group (**Figure 1A**). After 4 weeks administration, MGF reduced GLU, TG, and FFA levels in both of serum (**Figures 1B1–B3**) and liver (**Figures 1C1–C3**) at the dosage of 200 mg/kg/d. Compared with control group, fasting serum GLU, TG, and FFA levels were reduced by 49, 32, and 26%, respectively. While the fasting liver GLU, TG, and FFA levels were reduced by 45, 36, and 34%, respectively. MGF 100-mg/kg/d treated group only showed decreases effects on serum FFA, hepatic TG, and hepatic FFA levels.

Both of the MGF treated groups exerted a BW reducing tendency through the experiment period. The abdominal fat was significantly reduced while the ratio of interscapular BAT to WAT was remarkably increased in 200 mg/kg/d treated group (**Figure 1D**).

### MGF Inhibited Lipid Accumulation in KK-A^y^ Mice Liver

Liver is an important organ of lipid metabolism. Long-term excessive lipid accumulation usually results in hepatic steatosis. In control group, histologic examinations of liver in H&E and Oil Red O staining showed severe lipid accumulation indicated by the unstained vacuoles-like area under H&E staining and the red color area under the Oil Red O staining. Administration of MGF markedly alleviated hepatic triglyceride accumulation at dosage of 100 and 200 mg/kg/d (**Figure 1E**).

### MGF Administration Decreased Relative FFA Content in Mice Liver

**FIGURE 2:**
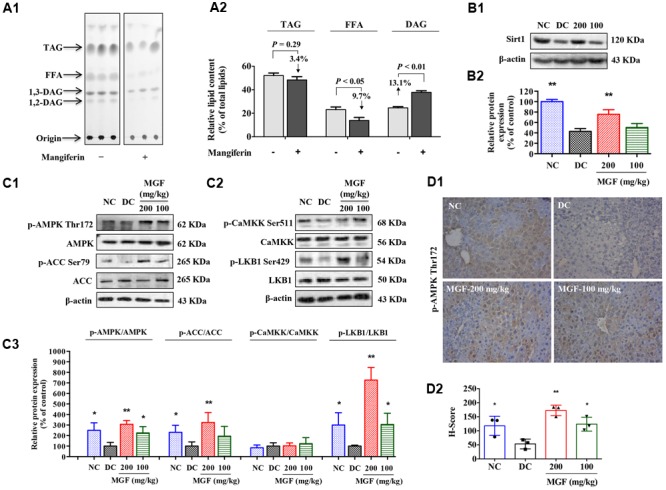
Mangiferin administration decreased hepatic lipid accumulation via SIRT1-AMPK activation. **(A)** Separation of hepatic lipid by thin-layer chromatography (TLC) analysis **(A1)**, and semi-quantification analysis by ImageJ software **(A2)**. Gray-scale values were represented by % of the total lipid from three separate mice in each group. **(B)** Protein expression of Sirt1 **(B1)** was suppressed but restored by MGF in a dose-dependent manner. Densitometric quantification of the protein expressions was measured by ImageJ software **(B2)**. Values are means ± SEM, ^∗∗^*p* < 0.01, ^∗^*p* < 0.05 versus DC group. **(C)** AMP-activated protein kinase (AMPK) activity **(C1)** and liver kinase B1 (LKB1) activity **(C2)** were suppressed but restored by MGF in a dose-dependent manner. Densitometric quantification of the protein expressions was measured by ImageJ software **(C3)**. Values are means ± SEM, ^∗∗^*p* < 0.01, ^∗^*p* < 0.05 versus DC group. **(D)** Expression of *p*-AMPK in the liver determined by immunohistochemical staining **(D1)** and quantitative analyzed by Histoscore system **(D2)**. Values are means ± SEM.

Liver TG homeostasis is a complex process that involves FFA flux to the liver, TG synthesis and lipogenesis. DAG, FFA, and TG ratio is important index for the development of hepatic TG metabolism disorder (Nassir et al., 2015). To investigate whether MGF affects the lipid composition in liver, total lipid extracts of fasted mice liver were analyzed by TLC. As shown in **Figures 2A1,A2**, there were no significant difference observed in relative TG content (% of total lipid content) between control group and MGF treated group. But relative FFA content (% of total lipid content) was decreased in 9.7% and DAG increased in 13.1%.

### The Hypolipidemic Effect of MGF Is Associated With the SIRT-1-AMPK Activation

SIRT-1/AMPK has previously been reported to play an important role in metabolic tissues as the energy sensor. To determine whether the MGF lipid lowering effect was associated with SIRT-1-AMPK signaling pathway, we examined the hepatic levels of SIRT-1, AMPK, and ACC. As shown in **Figures 2B,C**, AMPK/ACC phosphorylation and SIRT-1 expression were significantly increased in a dose-dependent manner in the MGF-treated mice liver, which was further confirmed by immunohistochemical staining of *p*-AMPK Thr172 (**Figure 2D**).

Activation of AMPK requires phosphorylation by upstream kinases, such as LKB1 and CaMKK (Shaw et al., 2004). Compared with control group, LKB1 phosphorylation level was significantly increased in MGF-treated mice liver, while no significant change was observed in CaMKK phosphorylation level (**Figure 2C**).

### MGF Reduced Hepatic Lipid Synthesis, Promoted Lipolysis and Fatty Acid Oxidation

**FIGURE 3:**
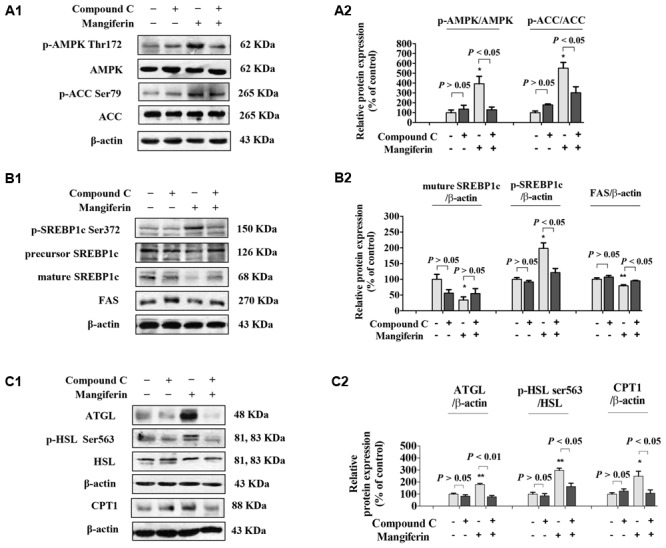
Mangiferin reduced hepatic lipid synthesis, promoted lipolysis, and increased fatty acid oxidation. **(A)** MGF-mediated AMPK activation was suppressed by Compound C in KK-A^y^ mice liver **(A1,A2)**. **(B)** MGF-mediated SREBP-1c processing and lipid synthesis suppression effects were inhibited by Compound C in KK-A^y^ mice liver **(B1,B2)**. **(C)** MGF-mediated lipolysis suppression effect and fatty acid oxidation activation effect were inhibited by Compound C in KK-A^y^ mice liver **(C1,C2)**. Densitometric quantification of protein expressions were measured by ImageJ software. Values are means ± SEM, ^∗∗^*p* < 0.01, ^∗^*p* < 0.05 versus DC group.

SREBP-1c is a key lipogenic transcription factor that preferentially regulates the lipogenesis. The activity of SREBP is reflected by the cleavage processing that the precursor (126 kDa) form undergoes sequential proteolytic processing to release the transcriptionally mature form (68 kDa) and is then migrated into the nucleus (Ferre and Foufelle, 2010). Activation of AMPK can phosphorylate SREBP-1c Ser372 to inhibit its activity and attenuate hepatic steatosis. Our results showed that MGF induced ACC phosphorylation could be decreased by Compound C co-treatment (**Figures 3A1,A2**), while the phosphorylation level of hepatic SREBP-1c was significantly increased by MGF to 1.98-fold, meanwhile, the accumulation of nuclear mature SREBP-1c was markedly reduced to 0.34-fold, and those effects were almost abolished by Compound C co-treatment. As expected, SREBP-1c target gene FAS showed the similar response to the MGF treatment (**Figures 3B1,B2**).

To further determine whether MGF also have a role in regulating lipolysis and fatty acid oxidation, and whether it is AMPK dependent, we then examined the protein expression level of ATGL, HSL, and CPT1 in mice liver treated with MGF and/or Compound C. Our results showed that MGF treatment significantly up-regulated ATGL and CPT1 expression by 1.8- and 2.5-fold, respectively. And the effects were completely abolished by Compound C co-treatment. The phosphorylation level of HSL was significantly increased by threefold, which was also completely suppressed by Compound C co-treatment. These results indicated that the lipid-lowering effect of MGF is LKB1-AMPK dependent, and associated with its function in inhibiting hepatic lipid synthesis, promoting lipolysis and fatty acid oxidation (**Figures 3C1,C2**).

### Metabolites Analysis of MGF

After oral administration of MGF for 4 weeks in rats, three xanthone metabolites were isolated from rat urine using column chromatography and preparative HPLC. Their structures were elucidated by chemical and spectroscopic methods (Supplementary Data Sheet 1), which were norathyriol (M1), 1,3,7-trihydroxy-6-methoxyxanthone (M2), and 1,7-dihydroxyxanthone (M3).

An UPLC-QTOF-MS method for the identification of MGF metabolites in liver was developed and optimized. From liver tissue, compound MGF, M1, M2, and M3 can be detected. Similar to reference report, liver distribution analysis result indicated that accumulation amount of MGF was less than its metabolites (Supplementary Data Sheet 1; Liu et al., 2011). These results indicated that not only original type of MGF, but also its metabolites maybe active constituents on liver lipid metabolism.

### MGF Metabolites Suppressed TG Accumulation in HepG2 Cells

**FIGURE 4:**
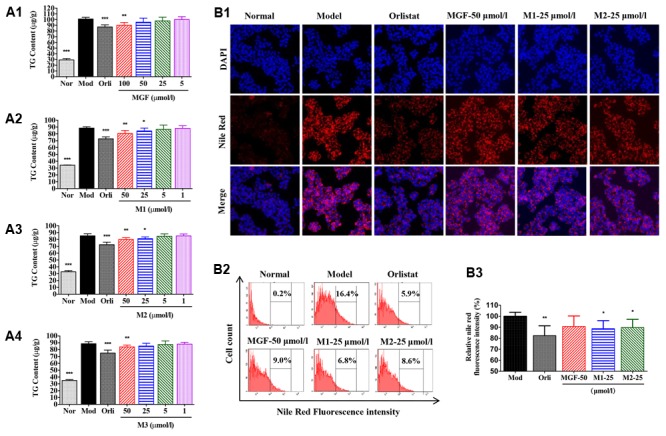
Mangiferin and its metabolites reduced lipid accumulation in SO-induced HepG2 cells. **(A)** MGF **(A1)** and its metabolites (M1, M2, and M3, **A2–A4**) had inhibitory effects on sodium oleate-induced TG accumulation in HepG2 cells. Values represent the mean ± SEM, ^∗∗∗^*p* < 0.001, ^∗∗^*p* < 0.01, ^∗^*p* < 0.05 versus the model (Mod) group. **(B)** After treatment, cells were fixed with formalin and stained with Nile red **(B1)** and analyzed by flow cytometry **(B2)** with quantitative assessment of the percentage of lipid accumulation **(B3)**. Values represent the mean ± SEM, ^∗∗^*p* < 0.01, ^∗^*p* < 0.05 versus the model (Mod) group.

To investigate the effects of MGF and its metabolites on lipid metabolism, HepG2 hepatocytes were challenged with 200 μmol/l SO. The results showed that SO treatment for 48 h significantly increased intracellular TG content (**Figures 4A1–A4**) as determined by intracellular TG assay and observed by Nile Red staining under inverted fluorescence microscope (**Figure 4B1**) or quantified by flow cytometry analysis (**Figures 4B2,B3**). Treatment of the three MGF metabolites reduced intracellular TG level in dose-dependent manners without influence on cell viability (data not shown). MGF metabolites suppressed TG accumulation more effectively than MGF at an equal molar concentration, among which M1 exhibited the most effective.

### M1 Reduce the TG Accumulation via AMPK-SREBP-1c Cascade in HepG2 Cells

**FIGURE 5:**
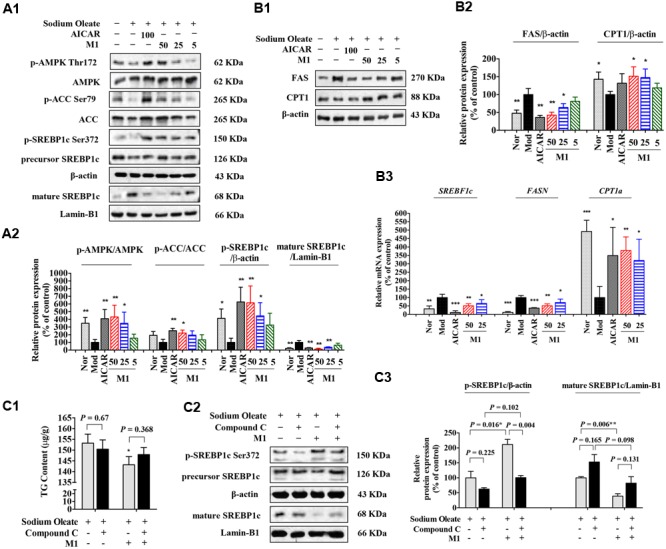
Norathyiol reduce the TG accumulation via AMPK-SREBP-1c cascade in HepG2 cells. **(A)** AMPK activity was increased and SREBP-1c processing was suppressed by M1 in a dose-dependent manner **(A1,A2)**. **(B)** The protein expression **(B1,B2)** and mRNA expression **(B3)** of fatty acid synthase (FAS) and carnitine palmitoyl transferase 1 (CPT1) were mediated by M1 in a dose-dependent manner. **(C)** Inhibitory effects of M1 on SO-induced TG accumulation and SREBP-1c processing in HepG2 cells were suppressed by Compound C. The intracellular TG contents were measured using a TG assay kit **(C1)**. Western blot analysis for mature SREBP-1c and p-SREBP-1c were conducted **(C2,C3)**. Values represent the mean ± SEM, ^∗∗^*p* < 0.01, ^∗^*p* < 0.05 versus the model (Mod) group.

Next, the effect of M1 on AMPK activity was determined. Our results showed that M1 phosphorylated AMPK and ACC in a dose-dependent manner. In parallel with the development of steatosis, the phosphorylation level of SREBP-1c was decreased and the level of mature form was significantly increased after challenged with SO for 12 h, which were dose-dependently reversed by M1 treatment. Concomitant with its augmented effect on the phosphorylation of AMPK and SREBP-1c, M1 suppressed the level of nuclear SREBP-1c, as did the AMPK activator AICAR (**Figures 5A1,A2**). Moreover, we also observed a strong regulatory effect of M1 on FAS and CPT1 expression in both mRNA and protein level (**Figure 5B**).

Indeed, in SO-induced TG accumulation HepG2 cells, M1 could significantly decrease the intracellular TG content, while the TG lowering effect of M1 was largely blocked by Compound C pre-treatment (**Figure 5C1**). Western blot analysis result indicated that M1 treatment significantly increased the level of *p*-SREBP-1c by more than twofold and decreased the expression level of SREBP-1c and its mature form (**Figures 5C2,C3**).

### MGF Metabolites Were Not AMPK Direct Activators

The above research results indicated that MGF and its metabolites exhibited regulation effect on lipid metabolism might through a pathway involving AMPK. To verify whether MGF and its metabolites are direct AMPK activators or not, a HTRF and radioactive filter assays were used. In this assay, another AMPK activator, A769662, at 50 nmol/l increased AMPKα1β1γ1 or AMPKα2β1γ1 activity by about two or fourfold, respectively. However, all the tested compounds showed no significant increasing activity on either AMPKα1β1γ1 or AMPKα2β1γ1 at 25 μmol/l (Supplementary Data Sheet 2). These results indicated that MGF and its metabolites were not direct activators of AMPK.

### The Effect of M1 on AMPK Activation Was Mediated by Regulating SIRT-1-LKB1 Signal Pathway and AMP/ATP Level

**FIGURE 6:**
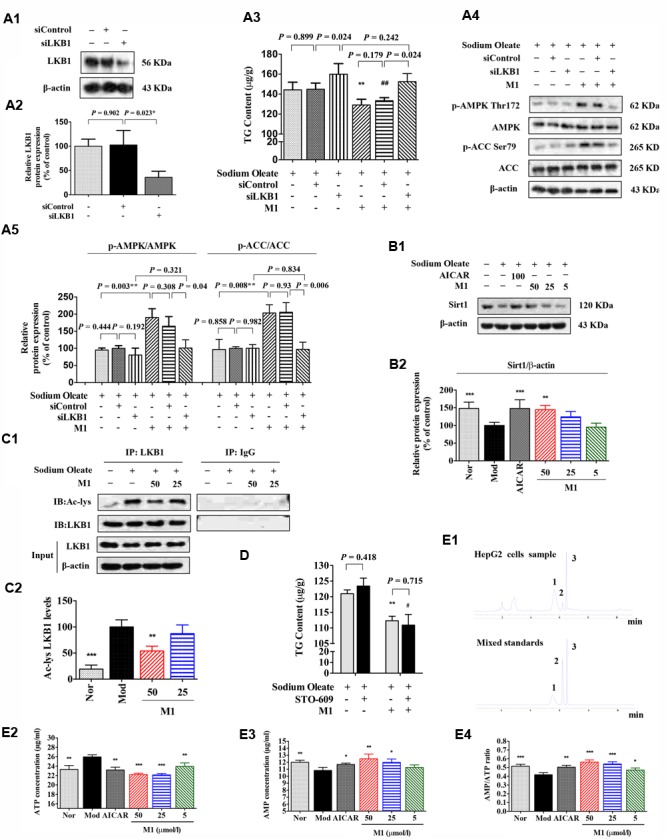
Effect of norathyiol on LKB1 and AMP level. **(A)** M1-mediated phosphorylation of AMPK was activated by LKB1. LKB1 protein expression after siRNA transfection was determined by western blot analysis **(A1,A2)**. The intracellular TG contents were measured using a TG assay kit **(A3)**. Western blot analysis for *p*-AMPK and *p*-ACC were conducted **(A4,A5)**. Densitometric quantification of protein expressions was measured by ImageJ software. Values represent the mean ± SEM, ^∗∗^*p* < 0.01 versus the model group, ##*p* < 0.01 versus the siControl group. **(B)** The protein expression of Sirt1 was increased by M1 in a dose-dependent manner **(B1,B2)**. **(C)** Immunoprecipitation of acetylated LKB1. Values represent the mean ± SEM, ^∗∗^*p* < 0.01, ^∗^*p* < 0.05 versus the model (Mod) group. **(D)** M1-mediated phosphorylation of AMPK was not activated by CaMKK. The intracellular TG contents were measured using a TG assay kit. Values represent the mean ± SEM, ^∗∗^*p* < 0.01 versus the model (Mod) group, #*p* < 0.05 versus the STO-609 group. **(E)** M1-mediated phosphorylation of AMPK was activated by the increase of ratio of AMP and ATP. The intracellular ATP and AMP contents were measured by HPLC analysis **(E1–E3)**. The result was analyzed by the ratio of AMP and ATP **(E4)**. Values represent the mean ± SEM, ^∗∗∗^*p* < 0.001, ^∗∗^*p* < 0.01, ^∗^*p* < 0.05 versus the model (Mod) group. 1 represents ATP, 2 represents ADP, 3 represents AMP.

Since MGF metabolites were not directly associated with AMPK, to further define the underlying mechanism of M1 function, AMPK upstream molecules were tested. Firstly, the siRNA knockdown efficiency under our experimental condition was determined. Western blot analysis showed that the expression of LKB1 was significantly reduced nearly by 60% after transfection (**Figures 6A1,A2**).

**Figure 6A3** showed that the TG lowering effect of M1 was completely blocked by LKB1 siRNA knockdown under the SO induction (**Figure 6A3**). In addition, Western blot analysis showed that the M1 failed to up-regulate the phosphorylation of AMPK and ACC after LKB1 knockdown (**Figures 6A4,A5**). However, in another SO-induced TG accumulation model, CaMKK inhibitor, STO-609 exhibited no obvious influence on the inhibitory effect of M1 (**Figure 6D**), which indicates CaMKK was not involved in M1 induced AMPK activation.

Hepatic SIRT-1 exerts an important role in hepatic fatty acid metabolism though regulating AMPK. SIRT-1 can directly deacetylates LKB1 and promote its movement from nuclear to cytoplasm where LKB1 activates AMPK (Hou et al., 2008; Lan et al., 2008). Therefore, we examined the SIRT-1 and acetylated LKB1 levels under the M1 treatment. As **Figures 6B,C** showed that M1 can significantly induce the SIRT-1 expression and decreased the acetylated LKB1 level in HepG2 cells.

Then, intracellular AMP and ATP levels were measured under the treatment with different concentrations of M1 in the SO model using HPLC method (**Figure 6E1**). The analysis results showed that M1 significantly decreased intracellular ATP content and increased AMP content compared with control group (**Figures 6E2,E3**) at the concentration of more than 25 μmol/l. The ratio of AMP and ATP was increased by M1 over the concentration of 5 μmol/l (**Figure 6E4**). The above results indicate that activation of AMPK by M1 was through regulating SIRT-1-LKB1 signal pathway and AMP/ATP level.

## Discussion

MGF is a natural xanthone distributed in a wide variety of plant. In this study, we found MGF attenuated excessive liver fat deposition and protected against hepatic steatosis using diabetic mice model. The novel points of our study include: (1) Norathyriol was the major active metabolite of MGF on hepatic lipid metabolism; (2) MGF and norathyriol regulates hepatic lipid metabolism through SIRT-1/LKB1 signaling mediated AMPK activation; and (3) MGF and norathyriol affect SREBP-1c activity through a post-translational modification.

AMPK is an intracellular energy sensor and regulator, which has been demonstrated to be closely related to insulin resistance and hepatic steatosis (Long and Zierath, 2006). AMPK phosphorylation leads to the phosphorylation and inactivation of ACC, and the inhibition of ACC by phosphorylated AMPK reduces substrate flow for FAS, thus decreasing the FAS activity (Browning and Horton, 2004; Smith et al., 2016). Our data shown MGF activated AMPK, blunted its downstream ACC and enhanced lipid oxidation, resulting in alleviated hepatic steatosis (**Figures 1, 2**). Similarly, MGF exerts a strong bioactive function in lipid oxidation and lipogenesis (Guo et al., 2011; Xing et al., 2014) partly through AMPK. Thus, we proposed that the anti-hepatic steatosis of MGF might be through inhibiting lipogenesis and enhancing lipid oxidation mediated by AMPK activation. However, there was no evidence to show whether AMPK was directly activated by MGF. In this study, our results suggested that MGF and its metabolites were not direct activators of AMPK, indicating MGF and its metabolites mediated AMPK activation might be dependent of its upstream molecules (Supplementary Data Sheet 2). Consistently, norathyriol failed to induce the phosphorylation of AMPK and ACC after LKB1 knockdown by siRNA (**Figure 6**).

SIRT-1 as a key metabolic sensor, is expressed in various metabolic tissues such as liver, skeletal muscle, adipose tissue, pancreas, and brain (Yang et al., 2006; Chalkiadaki and Guarente, 2012). Sirt1 and AMPK interplay each other. Both of them regulate essential cellular processes including energy metabolism and stress response (Caron et al., 2014). It is reported that SIRT-1 levels are inversely associated with NAFLD incidence (Deng et al., 2007; Mariani et al., 2015). Overexpression of SIRT-1 ameliorates high fat diet induced hepatic steatosis with enhanced energy expenditure and improved glucose tolerance (Pfluger et al., 2008), whereas hepatic deletion of SIRT-1 exhibit a significantly increased triglyceride accumulation in hepatocytes (Purushotham et al., 2009). MGF could increase the levels of SIRT-1 and AMPK phosphorylation in mice liver (**Figure 3**). In HepG2 cells, MGF metabolic norathyriol could induce SIRT-1 expression and significantly reduced LKB1 acetylation, followed by AMPK activation (**Figure 6**). Sirt-1/AMPK pathway activation can reduce lipid accumulation in obese mice liver and improve insulin resistance in diabetes mice. Thus, we suggested that both AMPK and SIRT-1 activation mediated by MGF was involved in its anti-hepatic steatosis effect. SIRT-1 deficiency would decrease TG hydrolysis by reducing ATGL expression (Chakrabarti et al., 2011). Here, we found that MGF induced hepatic ATGL, p-HSL, and CPT1 expression levels, which in turn promoted the lipolysis and fatty acid oxidation (**Figure 3**).

Sterol regulatory element binding proteins (SREBPs) are key lipogenic transcription factors on cellular lipid metabolism and homeostasis. Among isoforms of SREBPs, SREBP-1c is involved in fatty acid synthesis and energy storage (Shimano and Sato, 2017). It is reported that AMPK activation directly phosphorylated the Ser372 residue of SREBP-1c, resulted in suppression of SREBP-1c precursor cleavage and nuclear translocation, leading to the inhibition of SREBP-1c mediated lipogenesis (Li et al., 2011; Ha et al., 2016). However, evidence is still lacking to demonstrate whether MGF regulates post-translational modification of SREBP-1c. Our results demonstrated that oral administration of MGF in KK-A^y^ mice increased phosphorylation level of hepatic SREBP-1c, and reduced nuclear mature SREBP-1c and FAS levels in an AMPK-dependent manner (**Figure 2**). In addition, similar effect was observed in HepG2 cells treated with norathyriol (**Figure 5**). These findings suggested that MGF and its metabolite reduce lipogenesis and promote lipolysis through regulating SIRT-1/LKB1/AMPK/SREBP-1 signaling pathways, resulting in alleviation of hepatic steatosis.

From a drug metabolism perspective, as a xanthone carbon glycoside, low solubility of MGF in most aqueous and organic solvents causes difficulties for it passing through intestinal blockage and keep maintaining its original structure after liver metabolism. Previous studies by another group showed that the bioavailability of oral MGF was 1.2% in rats (Tian et al., 2016), implying that MGF might not be the final active compound regulating lipid metabolism. Using phytochemical methods, we isolated the metabolites of MGF from rat urine after long term oral administration. The main metabolite structure was identified as norathyriol by spectrographic methods. Interestingly, we found that MGF metabolites couldn’t be detected in either cell or cell culture medium after treated HepG2 cells with MGF, which suggested that MGF may be metabolized mainly through intestinal flora, then its metabolites were absorbed and transported to its site of action (Supplementary Data Sheet 3). Our results demonstrated MGF metabolite norathyriol shown more potent effect to decrease intracellular TG content, as well as regulating SREBP-1c activity in SO-induced TG accumulation HepG2 cells, compared with MGF. It is reported that norathyriol was a competitive inhibitor of protein tyrosine phosphatase 1B (Ding et al., 2014) and UDP-glucuronosyltransferase isoforms (Sun et al., 2017) to improve glucose homeostasis. In this study, we firstly reported the lipid lowering effect of norathyriol and its underlying mechanism in regulating hepatic lipid metabolism.

**FIGURE 7:**
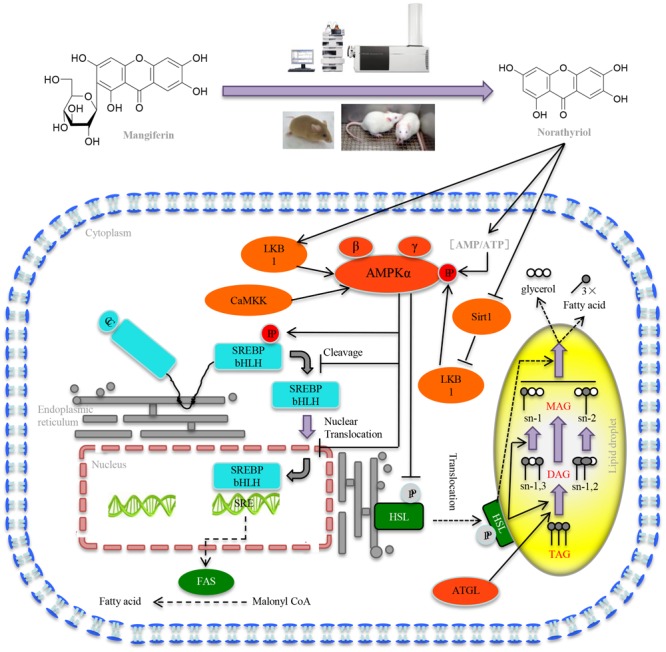
Mechanisms of MGF on liver lipid metabolism. MGF coverts to norathyriol by either intestinal flora or the metabolizing enzymes in liver, which increases the expression of Sirt1and phosphorylation of LKB1, as well as intracellular AMP/ATP level, leading to the activation of AMPK, promoting hepatic SREBP-1c phosphorylation level and reducing the level of nuclear mature SREBP-1c. Finally, it exerts the effects of inhibiting hepatic lipid synthesis, promoting lipolysis and increasing fatty acid oxidation.

Taken together our results demonstrated that MGF attenuated excessive liver fat deposition and protected against hepatic steatosis by suppressing SREBP-1c related lipogenesis, promoting lipolysis and fatty acid oxidation via regulating SIRT1/AMPK pathway (**Figure 7**). We showed its metabolites norathyriol exert a much stronger bioactivity in lipid metabolism, revealing the potential therapeutic use of norathyriol in treatment of NAFLD.

## Author Contributions

JL, ML, YZ, and TW contributed to experimental design. JL, ML, HY, WW, LH, QC, JR, and SW contributed to the acquisition and analysis of data. ML, HY, and WW reviewed the manuscript. YZ and TW obtained the funding. JL, ML, and TW wrote the manuscript.

## Conflict of Interest Statement

The authors declare that the research was conducted in the absence of any commercial or financial relationships that could be construed as a potential conflict of interest. The reviewer EL and handling Editor declared their shared affiliation.

## Abbreviations

ACC: acetyl CoA carboxylase
AMPK: AMP-activated protein kinase
ATGL: adipose triglyceride lipase
BAT: brown adipose tissue
BW: body weight
CaMKK: calmodulin-dependent protein kinase kinase
CPT1: carnitine palmitoyl transferase 1
DAG: diacylglycerol
FAS: fatty acid synthase
FFA: Free fatty acid
GLU: glucose
H&E: hematoxylin and eosin
HPLC: high performance liquid chromatography
HSL: hormone sensitive lipase
HTRF: homogeneous time resolved fluorescence
LKB1: liver kinase B1
MGF: mangiferin
siRNA: small interfering RNA
SIRT-1: sirtuin-1
SO: sodium oleate
SREBP-1c: sterol regulatory element binding protein-1c
TG: triglycerides
TLC: thin-layer chromatography
WAT: white adipose tissue
