# Supplementary material for: Mangiferin Improves Hepatic Lipid Metabolism Mainly Through Its Metabolite-Norathyriol by Modulating SIRT-1/AMPK/SREBP-1c Signaling
Source: Front Pharmacol. 2018 Mar 7;9:201. doi: 10.3389/fphar.2018.00201 (PMC5850072; doi:10.3389/fphar.2018.00201)

**Supplement 1. Urine metabolites isolation and structure identificantion, Liver metabolites analysis of oral administration of MGF in rat.**

**1. Experimental**

**1.1. Chemicals**

HPLC grade acetonitrile and acetic acid were purchased from Merck Technologies Inc. (Darmstadt, Germany), and Tedia Company Inc. (Fairfield, OH, USA). Deionized water was obtained from a Millipore Milli-Q water system (Bedford, MA, USA). All other reagents were of analytical purity.

Mangiferin (MGF) for dosing was purchased from Zhongxin Pharmaceutical Group Co., Ltd. (Tianjin, China)，purity of which was more than 90% determined by a high performance liquid chromatography (HPLC) method. The standard MGF (99%, purity) for reference was purchased from the National Institute for Control of Pharmaceutical and Biological Products (Beijing, China).

**1.2. Animals and treatment**

SD rats (220 ± 10 g; Vital River Laboratory Animal Technology Co. Ltd., Beijing, China) were kept in an environmentally controlled breeding room for 7 days before experimentation, fed with standard laboratory food and water.

***Urine sample collection for metabolites isolation of oral administration of Mangiferin***

In order to isolate the metabolites, 30 rats were used for collecting urine sample. Rats were housed in metabolic cages (Suzhou Fengshi Laboratory Animal Equipment Co.,Ltd, China) and orally administrated of MGF at a dose of 100 mg/kg/d every day for 30 consecutive days. Urine samples were collected each day and combined for the further compounds extractions. After been collected, urine sample was immediately filtered and the filtrate was stored at -20°C with 0.01% sodium azide.

***Liver metabolites analysis for oral administration of Mangiferin***

Nine rats were administrated of Mangiferin at a dose of 100 mg/kg/d in 1% arabic gum water solution for 9 days. The administration volume was 1 mL/100 g. Simultaneously, three rats were set as normal control and treated with the same volume of water.

**1.3 Extraction and isolation of metabolites from urine**

The collected urine samples were combined together. Same volume of each urine fraction was subjected to D101 macroporous resin chromatography column (CC) and eluted with H_2_O and 95% EtOH stepwise. The 95% EtOH eluate (5 g) was centrifuged in MeOH at 14000 r/min for 15 min. Then the supernatant was isolated by Sephadex LH-20 CC (MeOH) to yield seven fractions (Fr. 1-Fr. 7). Fraction 6 (132.0 mg) was purified by preparative HPLC (PHPLC) [MeOH-H_2_O (50:50, v/v) + 1% HAc] to gain 1,3,6,7-tetrahydroxyxanthone (**1**, 7.9 mg), 1,3,7-trihydroxy-6-methoxyxanthone (**2**, 2.5 mg). Fraction 7 (139.6 mg) was prepared by PHPLC [MeOH-H_2_O (80:20, v/v) + 1% HAc] to give 1,7-dihydroxyxanthone (**3**, 5.0 mg).

**1.4 UHPLC/ESI Q-Orbitrap MS analysis of mangiferin and its metabolites in liver**

*1.3.1 Preparation of standard solutions*

Standard test solutions of mangiferin, 1,3,6,7-tetrahydroxyxanthone, 1,3,7-trihydroxy-6-methoxyxanthone, and 1,7-dihydroxyxanthone were prepared in MeOH at a concentration of approximately 5 ng/mL. The standard test solutions were stored at 4 °C in darkness and brought to room temperature before use.

An aliquot of 1.0 g of accurately weighed fine powder of UR stems and hooks was immersed in 30 mL 70 % aqueous methanol (v/v) and extracted on a water bath at 40 °C with ultrasound assistance for 30 min. The extract was centrifuged at 6000 rpm for 10 min. The supernatant was then filtered through a 0.2-μm PTFE microporous membrane (Agilent Technologies, Santa Clara, CA, USA) to prepare the test solutions. Test solutions of 14 alkaloid compounds were prepared in methanol at a concentration of 0.10 mg/mL.

*1.3.2 Preparation of liver sample*

Twelve hours after final administration, liver sample were collected after hepatic perfusion with phosphate-buffered saline. The collected organs were flash frozen by liquid nitrogen immediately and all the samples were stored at -80 °C.

Liver sample was ultrasound homogenized with 4 volumes of acetonitrile-acetic acid (9:1, v/v). The supernatant was separated after been votexed and centrifuged at 10,000×g for 10 min. The above extracting solution was dried under nitrogen gas. The residues were reconstituted in 100 µL methanol and centrifuged at 14,000 g for 10 min prior to analysis. The normal control sample was prepared with the same method.

*1.3.3 Chromatographic and mass spectrometry conditions*

The analysis was performed on an ultra-high performance liquid chromatography combining with quadrupole Orbitrap high-resolution mass spectrometry (UHPLC/ESI Q-Orbitrap MS). Chromatographic separation of the metabolites were achieved on a reversed phase ACQUITY UPLC BEH C18 column (2.1 mm × 100 mm, 1.7 μm, Waters Milford, MA, USA) at 35°C with the flow rate 0.4 mL/min. Mobile phase consisting of water containing 0.1% acetic acid (A) and acetonitrile (B) used a gradient program as follows: 5%-67% B at 0–9.5 min, linearly gradient to 100% B in 13 min, hold for 2 min, then linearly gradient to 5% B at 15.5 min, hold for 2 min. Sample temperature was set to be 4°C. An aliquot of 3 μL of the purified sample was injected automatically into the HPLC system for LC-MS analysis.

The ESI source parameters were set as follows: ion spray voltage 3.2 kV, capillary temperature 350 °C, ion source heater temperature 300 °C, sheath gas (N_2_) 40 arbitrary units, auxiliary gas (N_2_) 10 arbitrary units, and a normalized collision energy (NCE) of 35 V was used. The Orbitrap analyzer scanned the mass range from *m/z* 150 to 1500. Monitoring time was 0-9.5 min. Detection was obtained by full mass-Single Ion Monitoring (SIM)/Targeted mode. The MS data were recorded in the profile and centroid formats, respectively. Data recording and processing were performed using the Xcalibur 4.0 software (Thermo Fisher Scientific, Inc., Waltham, MA, USA). The accuracy error threshold was fixed at 2 ppm.

**2. Results**

**2.1. Structure identification**

Metabolite 1: 1,3,6,7-tetrahydroxyxanthone (Norathyriol), yellow powder. HRESI-ESI-Q-Orbitrap MS: Negative-ion mode *m/z* 259.02478 [M–H]^–^ (calcd for C_13_H_7_O_6_, 259.02481). ^1^H NMR (500 MHz, DMSO-*d*_6_): *δ* 6.15 (1H, br. s, H-2), 6.32 (1H, br. s, H-4), 6.83 (1H, s, H-5), 7.36 (1H, s, H-8), 13.22 (1H, br. s, 1-OH). ^13^C NMR (125 MHz, DMSO-*d*_6_): *δ* 162.5 (C-1), 97.6 (C-2), 164.5 (C-3), 93.5 (C-4), 157.2 (C-4a), 102.4 (C-5), 154.6 (C-6), 143.9 (C-7), 107.6 (C-8), 111.3 (C-8a), 178.7 (C-9), 101.4 (C-9a), 151.0 (C-10a).

Metabolite 2: 1,3,7-trihydroxy-6-methoxyxanthone, yellow powder. HRESI-ESI-Q-Orbitrap MS: Negative-ion mode 273.04147 [M–H]^–^ (calcd for C_14_H_9_O_6_, 273.04046). ^1^H NMR (500 MHz, DMSO-*d*_6_): *δ* 6.04 (1H, br. s, H-2), 6.21 (1H, br. s, H-4), 6.42 (1H, s, H-5), 7.18 (1H, s, H-8), 13.72 (1H, br. s, 1-OH), 3.78 (3H, s, 6-OCH_3_). ^13^C NMR (125 MHz, DMSO-*d*_6_): *δ* 162.3 (C-1), 97.5 (C-2), 164.8 (C-3), 93.4 (C-4), 156.8 (C-4a), 101.9 (C-5), 148.7 (C-6), 154.2 (C-7), 102.6 (C-8), 105.9 (C-8a), 177.1 (C-9), 100.8 (C-9a), 163.9 (C-10a), 55.2 (6-OCH_3_).

Metabolite 3: 1,7-dihydroxyxanthone, yellow powder. HRESI-ESI-Q-Orbitrap MS: Negative-ion mode 227.03453 [M–H]^–^ (calcd for C_13_H_7_O_4_, 227.03498). ^1^H NMR (500 MHz, DMSO-*d*_6_): *δ* 6.79 (1H, br. d, *ca*. *J* = 9 Hz, H-2), 7.71 (1H, dd, *J* = 8.5, 8.5 Hz, H-3), 7.05 (1H, *ca*. *J* = 9 Hz, H-4), 7.55 (1H, d, *J* = 9.0 Hz, H-5), 7.38 (1H, dd, *J* = 3.0, 9.0 Hz, H-6), 7.45 (1H, d, *J* = 3.0 Hz, H-8). ^13^C NMR (125 MHz, DMSO-*d*_6_): *δ* 160.8 (C-1), 109.5 (C-2), 137.1 (C-3), 107.0 (C-4), 155.7 (C-4a), 119.3 (C-5), 125.5 (C-6), 154.0 (C-7), 107.7 (C-8), 120.3 (C-8a), 107.8 (C-9a), 181.5 (C-9), 149.2 (C-10a). On the basis of 1D- and 2D-NMR (^1^H-^1^H COSY, HSQC, HMBC) spectra analysis, the structure was identified as 1,7-dihydroxyxanthone.

**2.2. Qualitative analysis of mangiferin and its metabolites in liver**

A target-sim mode was used to accomplish the qualitative analysis of mangiferin and its metabolites in liver tissue. As result, an UHPLC/ESI Q-Orbitrap MS method was established and successfully applied to prove the accumulation of mangiferin and its metabolites 1, 2, 3 in liver tissue.


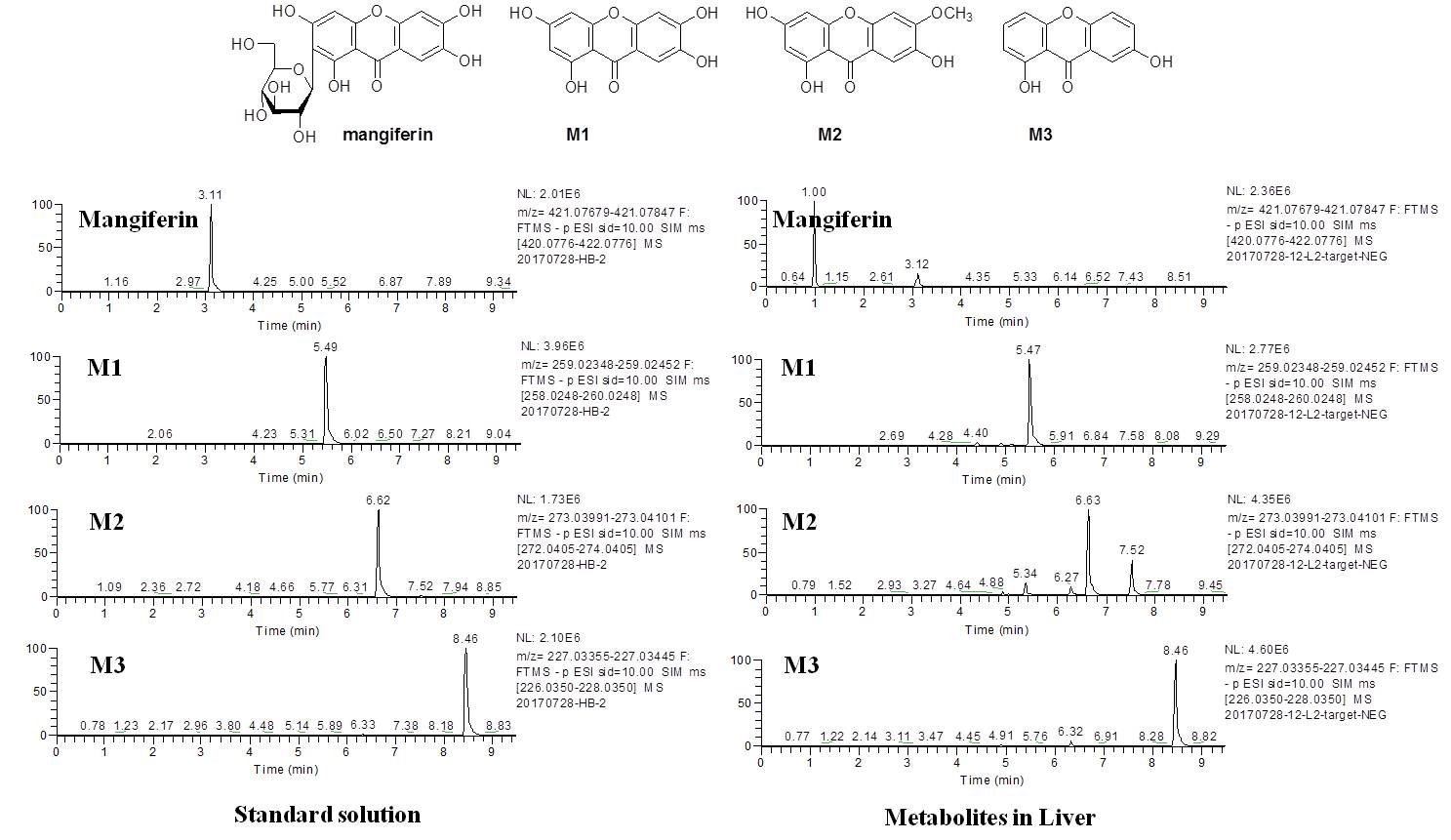

Supplement: Supplementary file 1 [file Data_Sheet_1.DOCX]
