# Supplementary material for: Mangiferin Improves Hepatic Lipid Metabolism Mainly Through Its Metabolite-Norathyriol by Modulating SIRT-1/AMPK/SREBP-1c Signaling
Source: Front Pharmacol. 2018 Mar 7;9:201. doi: 10.3389/fphar.2018.00201 (PMC5850072; doi:10.3389/fphar.2018.00201)
Supplement: Supplementary file 2 [file Data_Sheet_2.DOCX]

**Supplement 2**

***Activation test of AMPK in vitro by*** ***homogeneous time resolved fluorescence (HTRF) assay***

AMPK activity was measured following a literature described protocol([Dong et al., 2016](#_ENREF_1)). Briefly, AMPK protein was completely phosphorylated by incubation with CaMKKα at 30 °C for 4 h. The AMPK activation activity was tested with the STK substrate, 1-biotin, XL-665 and STK-Antibody of HTRF^®^ KinEASE™-STK1 Kit. The reaction was carried out in a reaction volume of 10 μl containing 32 mmol/l Tris–HCl (pH 7.5), 4 mmol/l MgCl_2_, 0.8 mmol/l DTT, 160 μmol/l substrate-1 peptide and 4 μmol/l ATP. The reaction was initiated by addition of 1.6 nmol/l recombinant AMPKα1β1γ1 or AMPKα2β1γ1 into the well, and incubation for 45 min at 30 °C. The reaction was terminated by addition of detection reagent containing 57.5 nmol/l XL-665 and STK-Antibody labeled with Eu^3+^-Cryptate. The fluorescence was measured at 615 nm and 665 nm. A ratio calculated (665/615) represented the AMPK activity.

HTRF assay for MGF and its metabolites against AMPKα1β1γ1 (A1) and AMPKα2β1γ1 (A2). 50 nmol/l AMPK activator, A769662, or 25 μmol/l MGF, M1, M2, and M3 were incubated with HTRF reagent as described in Section 2.10, respectively. The ratio of the fluorescence at 665 nm and 615 nm represented the AMPK activity. Values represent the mean ± S.E.M.

**Reference**

.Dong, C., Xie, Z., Yu, Y., Li, J., Liu, J., Li, J., et al. (2016). Discovery, synthesis, and structure-activity relationships of 20S-dammar-24-en-2alpha,3beta,12beta,20-tetrol (GP) derivatives as a new class of AMPKalpha2beta1gamma1 activators. *Bioorg Med Chem* 24(12)**,** 2688-2696. doi: 10.1016/j.bmc.2016.04.034.
