# Supplementary material for: Mangiferin Improves Hepatic Lipid Metabolism Mainly Through Its Metabolite-Norathyriol by Modulating SIRT-1/AMPK/SREBP-1c Signaling
Source: Front Pharmacol. 2018 Mar 7;9:201. doi: 10.3389/fphar.2018.00201 (PMC5850072; doi:10.3389/fphar.2018.00201)
Supplement: Supplementary file 3 [file Data_Sheet_3.DOCX]

**Supplement 3. UHPLC/ESI Q-Orbitrap MS analysis of mangiferin and its metabolites in Cells**

*1.1 Preparation of standard solutions,*

Standard test solutions of mangiferin, 1,3,6,7-tetrahydroxyxanthone, 1,3,7-trihydroxy-6-methoxyxanthone, and 1,7-dihydroxyxanthone were prepared in MeOH at a concentration of approximately 5 ng/mL. The standard test solutions were stored at 4 °C in darkness and brought to room temperature before use.

*1.2 Preparation of cell sample and cell culture medium sample*

Cell sample was ultrasound homogenized with 3 volumes of acetonitrile-acetic acid (3:1, v/v). The supernatant was separated after been vortexed and centrifuged at 14,000×g for 10 min. The above extracting solution was dried under nitrogen gas. The residues were reconstituted in 200 µL methanol and centrifuged at 14,000 g for 10 min prior to analysis. The normal control sample was prepared with the same method.

cell culture medium sample was vortexed and centrifuged at 14,000×g for 10 min. The supernatant was dried under nitrogen gas. The residues were reconstituted in 200 μL methanol and centrifuged at 14,000 g for 10 min prior to analysis.

*1.3 Chromatographic and mass spectrometry conditions*

The analysis was performed on an ultra-high-performance liquid chromatography combining with quadrupole Orbitrap high-resolution mass spectrometry (UHPLC/ESI Q-Orbitrap MS). Chromatographic separation of the metabolites were achieved on a reversed phase ACQUITY UPLC BEH C18 column (2.1 mm × 100 mm, 1.7 μm, Waters Milford, MA, USA) at 35°C with the flow rate 0.4 mL/min. Mobile phase consisting of water containing 0.1% acetic acid (A) and acetonitrile (B) used a linearly gradient program as follows: 5%-67% B at 0–9.5 min, linearly gradient to 100% B in 13 min, hold for 2 min, then linearly gradient to 5% B at 15.5 min, hold for 2 min. Sample temperature was set to be 4°C. An aliquot of 3 μL of the purified sample was injected automatically into the HPLC system for LC-MS analysis.

The ESI source parameters were set as follows: ion spray voltage 3.2 kV, capillary temperature 350 °C, ion source heater temperature 300 °C, sheath gas (N_2_) 40 arbitrary units, auxiliary gas (N_2_) 10 arbitrary units, and a normalized collision energy (NCE) of 35 V was used. The Orbitrap analyzer scanned the mass range from m/z 150 to 1500. Monitoring time was 0-9.5 min. Detection was obtained by full mass-Single Ion Monitoring (SIM)/Targeted mode. The MS data were recorded in the profile and centroid formats, respectively. Data recording and processing were performed using the Xcalibur 4.0 software (Thermo Fisher Scientific, Inc., Waltham, MA, USA). The accuracy error threshold was fixed at 2 ppm.

**2. Results**

An ultra-high performance liquid chromatography combining with quadrupole Orbitrap high-resolution mass spectrometry (UHPLC/ESI Q-Orbitrap MS) method was established and successfully applied to prove the accumulation of mangiferin and its metabolites 1, 2, 3 in cell. A target-sim mode was used to accomplish the qualitative analysis of mangiferin and its metabolites in cell. Mangiferin and its metabolites 1, 2, 3 were prepared to be the standard references. Only mangiferin can be detected from cell and cell culture medium, the metabolites couldn't be found in either cell or cell culture medium after treated the cell with mangiferin.

**Mangiferin in blank medium:**

**Mangiferin in medium (50 μM Mangiferin):**

**Mangiferin in medium (100 μM Mangiferin):**

**Mangiferin in cell culture medium (cell + culture medium, 50 μM Mangiferin):**

**Mangiferin in cell culture medium (cell + culture medium, 100 μM Mangiferin):**

**Mangiferin in cells (cell + culture medium, 50 μM** **Mangiferin):**

**Mangiferin in cells (cell + culture medium, 100 μM** **Mangiferin):**

**Mangiferin in standard test solution:**
